# Aberrant Methylation of *RASSF1A* Closely Associated with HNSCC, a Meta-Analysis

**DOI:** 10.1038/srep20756

**Published:** 2016-02-09

**Authors:** Rui-Wei Meng, Yun-Cheng Li, Xiong Chen, Yang-Xin Huang, Hao Shi, Dan-Dan Du, Xun Niu, Cheng Lu, Mei-Xia Lu

**Affiliations:** 1 Department of Epidemiology and Biostatistics, and the Ministry of Education Key Lab of Environment and Health, School of Public Health, Tongji Medical College, Huazhong University of Science and Technology, Wuhan, Hubei, China; 2 Department of Otolaryngology, Union Hospital, Tongji Medical College, Huazhong University of Science and Technology, Wuhan, Hubei, China; 3 Department of Epidemiology and Biostatistics, College of Public Health, University of South Florida, Tampa, FL, USA; 4 Department of Anatomy, Medical College of Nanchang University, Nanchang, Jiangxi, China

## Abstract

The RAS association domain family protein 1a (*RASSF1A*), a tumor suppressor gene at 3p21.3, plays a very important role in various cancers, including the head and neck squamous cell carcinoma (HNSCC). Hypermethylation of CpG islands in the *RASSF1A* promoter region contribute to epigenetic inactivation. However, the association between *RASSF1A* promoter methylation and HNSCC remains unclear and controversial. Therefore, a meta-analysis was performed in the study to identify the association. We identified the eligible studies through searching PubMed, EMBASE, Web of Science, and China National Knowledge Infrastructure (CNKI) databases with a systematic searching strategy. The information on characteristics of each study and prevalence of *RASSF1A* methylation were collected. Pooled odds ratios (ORs) with corresponding confidence intervals (CIs) were calculated. Meta-regression was performed to analyze heterogeneity and funnel plots were applied to evaluate publication bias. A total of 550 HNSCC patients and 404 controls from twelve eligible studies were included in the meta-analysis. Overall, a significant association was observed between *RASSF1A* methylation status and HNSCC risk under a random-effects model (OR = 2.93, 95% CI: 1.58–5.46). There was no significant publication bias observed. The meta-analysis suggested that there was a significant association between aberrant *RASSF1A* methylation and HNSCC.

Head and neck cancer is the sixth most common cancer worldwide accounting for approximately 6% of all newly diagnosed malignancies. HNSCC makes up over 90% of head and neck cancer, and commonly arises from the mucosal lining in this region^1^. Epidemiological data demonstrates that heavy smoking and alcohol consumption contribute to HNSCC tumorigenesis^2^. Human papillomavirus (HPV) can also implicate the increased incidence of HNSCC in the United States^3^. Despite the advances in therapy, the overall survival rates of HNSCC have not improved significantly over the past several decades and more than 50% of patients have experienced local relapse and distant metastasis^4^. Early diagnosis of HNSCC might improve its prognosis, but it is usually not detected in the early stages of HNSCC. Therefore, the efforts to identify novel molecular predictors for HNSCC are instrumental for early diagnosis in the early stage of cancer development.

DNA methylation of cytosine-guanosine dinucleotides (CpG) islands within the promoter region of genes is an alternative mechanism of gene inactivation to gene deletion or mutation. Teschendorff^5^ observed that invasive cancers displayed increased DNA methylation at the risk CpG sites in contrast to normal tissue, but lower levels in contrast to pre-cancerous lesions. This revealed that aberrant DNA methylation of risk CpG loci was prior to the onset of cancer, indicating that epigenetic diversity in normal cells increased the risk of cancer. Aberrant DNA methylation is frequently considered to be critical in the early stage of cancer development, including HNSCC^6^. Previous studies had investigated the association between hypermethylation of tumor suppressor genes and HNSCC and evaluated the value of them as potential biomarkers of HNSCC^7^,^8^,^9^,^10^,^11^,^12^. *RASSF1A*, a kind of tumor suppressor gene, is one of eight isoforms of *RASSF1* which is involved in cell cycle control, microtubule stabilization, cellular adhesion and motility as well as apoptosis^13^. Epigenetic inactivation of *RASSF1A* by hypermethylation is originally described in lung and breast cancer^14^. Since then, it has emerged that *RASSF1A* is one of the most frequently hypermethylated genes so far described and was reported as a prognostic indicator in renal cell carcinoma, non-small cell lung cancer, neuroblastoma, endometrial cancer and breast cancer^15^,^16^,^17^,^18^,^19^,^20^,^21^. Furthermore, hypermethylation of *RASSF1A* within promoter CpG islands is frequently observed in the HNSCC cell lines^22^. All of these findings indicate that *RASSF1A* might play an important role in the development of HNSCC.

To date, a number of studies have investigated the association between aberrant methylation of *RASSF1A* and HNSCC through a comparison of the methylation prevalence of *RASSF1A* between cancerous tissues and controls. However, the obtained results of these studies are inconclusive and inconsistent^23^,^24^. Therefore, we conducted a meta-analysis of 12 published studies to conclude the association.

## Results

### Study characteristics

**Figure 1:**
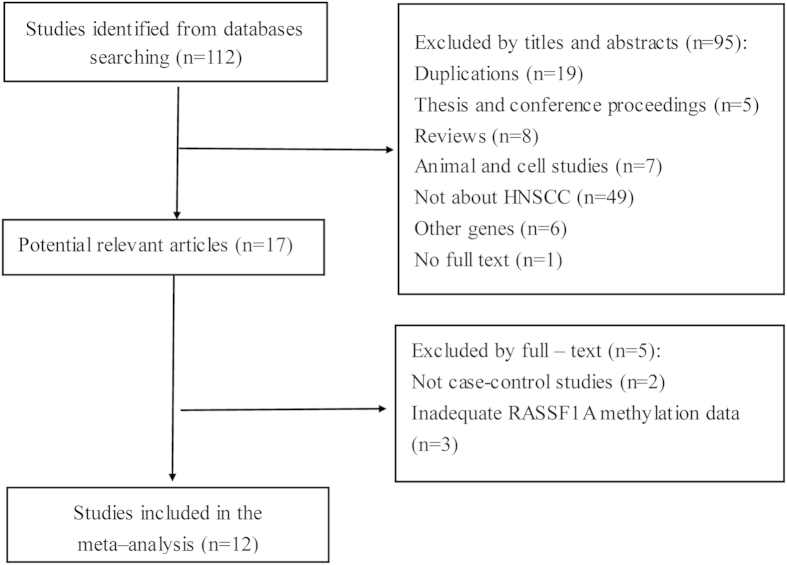
Selection of studies in the meta-analysis.

In total, the electronic search strategy initially identified 112 potentially relevant studies. Firstly, these potentially relevant studies were screened for inclusion based on their titles and abstracts. As a result, 19 duplications and 76 studies (four thesis, one conference proceeding, eight reviews, two animal studies, five cell lines, 49 not about HNSCC, six without *RASSF1A* and one without full text) were excluded. The remaining 17 citations were retrieved for full-text assessment. Upon the assessment, two articles which were not case-control studies and three articles with inadequate *RASSF1A* methylation data were excluded. Figure 1 showed the whole process of study selection and exclusion, with specification of reasons. Lastly, 12 studies, published between 2002 and 2012 with 18 to 111 cases, met the inclusion criteria and were included in our meta-analysis. The individual characteristics of the 12 included studies are summarized in Table 1.

The meta-analysis consisted of 550 cases of HNSCC tissues and 404 controls, with a total sample size of 954. Among the 12 included studies, the study populations were Caucasians in eight articles^22^,^25^,^26^,^27^,^28^,^29^,^30^,^31^ and Asians in four articles^23^,^24^,^32^,^33^. A total of nine studies conducted methylation-specific polymerase chain reaction (MSP) to assess the gene methylation status. Three articles used quantitative methylation-specific polymerase chain reaction (Q-MSP), bisulfite sequencing PCR (BSP) and methylation sensitive restriction analysis (MSRA) respectively to evaluate the *RASSF1A* methylation in cases and controls. The genomic location of the analyzed regions of eight studies included was the promoter. The genomic location of the analyzed regions of the remaining four articles was the CpG islands of the promoter. Hogg^34^ analyzed the methylation status of CpG islands in the promoter region of *RASSF1A* from LCTSGR1 at 3p21.3 in the HNSCC patients. 11 of the articles were published in English, and 1 was published in Chinese. The specimens were cancerous tissues of HNSCC cases and non-cancerous tissues of controls. The control group was comprised of HNSCC patients, benign disease patients and healthy volunteers.

### Meta-analysis results

**Figure 2:**
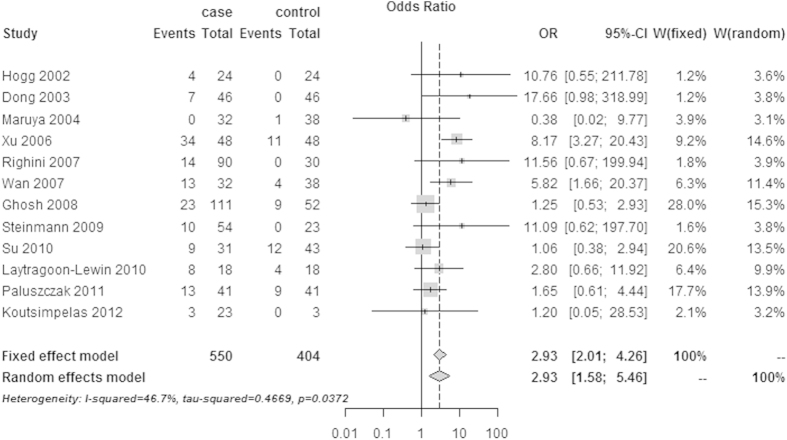
The estimates for *RASSF1A* methylation frequency associated with HNSCC in the meta-analysis.

The pooled ORs and corresponding ORs 95% CIs for the association between *RASSF1A* promoter methylation and HNSCC were shown in Fig. 2. A random-effects model was employed because a significant heterogeneity was observed among 12 included studies by the χ^2^-based Cochran Q statistic test and *I*^2^ statistics (*I*^*2*^ = 46.7%, Q = 20.65, *P* = 0.0372). In the overall meta-analysis, the *RASSF1A* promoter methylation was significantly associated with HNSCC, with a combined OR of 2.93 (95% CI: 1.58–5.46) under the random-effects model.

### Meta-regression analysis and subgroup analysis

A significant heterogeneity was found among the studies. Therefore we conducted a meta-regression to explore the source of heterogeneity with restricted maximum likelihood method (REML method^35^). Based on previous studies, we assumed that the heterogeneity might arise from the ethnicity, control types, age of patients, *RASSF1A* methylation detection methods, case sample size, HPV infection status, gender proportion smoking status and histology types. However, only the data about ethnicity, control types, methods of *RASSF1A* methylation detection and case sample size were collected completely. Then, we conducted a multiple regression model with following four variables: races (Asians and Caucasians), control types (autologous control heterogeneous control), methods used to *RASSF1A* methylation detection (MSP, Q-MSP and BSP) and case sample size (≥40 and <40). According to the result of the meta-regression analysis result, all 95% confidence intervals included 0 for the coefficients which indicated that none of the variables can explain the heterogeneity between-studies in Table 2. Furthermore, we performed a subgroup analysis of those variables in Table 3. The ORs were 10.76 (95% CI: 0.55–211.78) in the BSP group, 1.06 (95% CI: 0.38–2.94) in the Q-MSP group, and 3.30 (95%CI: 2.17–5.01) in the MSP group (MSRA was classified as MSP group) under the fixed-effects model, respectively. The heterogeneity did not change significantly in the subgroup analysis of detection methods. Similar results on the change of heterogeneity were found in other subgroup analysis.

### Sensitivity Analysis

**Figure 3:**
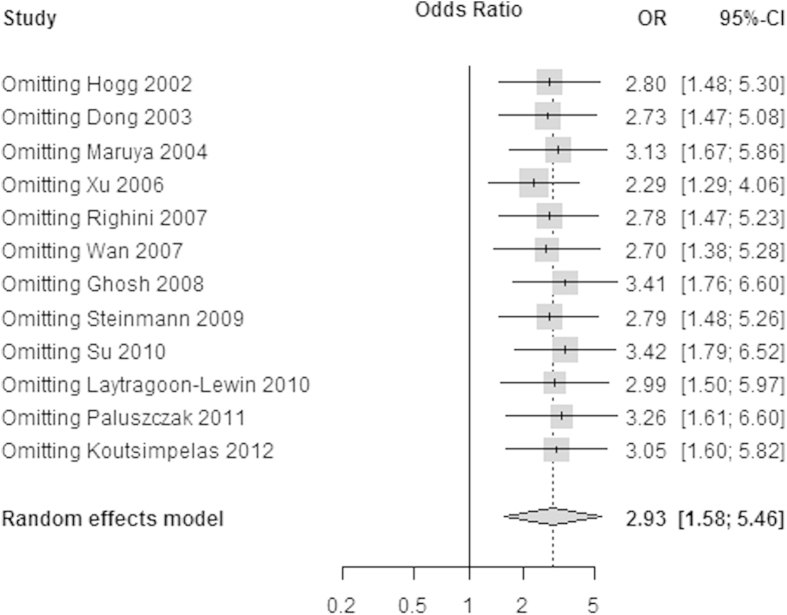
The sensitivity analysis by omitting a single study under the random-effects method.

Sensitivity analysis was performed by omitting a single study under the random-effects model. The results of sensitivity analysis showed that the pooled ORs ranged from 2.29 (95% CI: 1.29–4.06) to 3.42 (95% CI: 1.79–6.52). This demonstrated that none of the studies dramatically influenced the pooled ORs in Fig. 3. The REML method was used to estimate the variance between studies.

### Publication Bias

**Figure 4:**
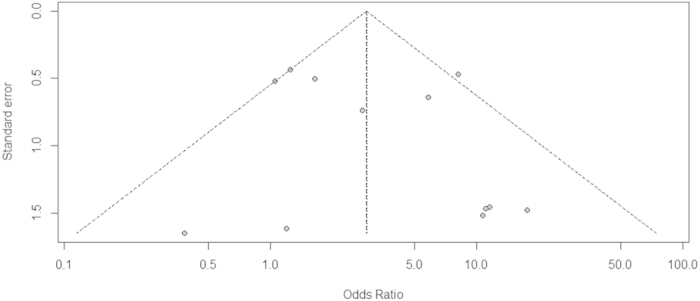
The Begg’s funnel plot for assessment of publication bias in the meta-analysis (each study is represented by a point).

We performed a Begg’s funnel plot^36^ and Peter test^37^ to assess the publication bias of the included studies. The shape of the Begg’s funnel plot showed no obvious asymmetry and absence of symmetry indicated publication bias (Fig. 4). No publication bias was detected by Peter test (*P* = 0.73) and Begg’s rank correlation test^36^ (*P* = 0.87), respectively. Furthermore, the fail-safe number^38^ was applied to evaluate the publication bias. If this number was relatively large to the number of observed studies, we could feel fairly confident in the summary conclusions. The fail-safe number (*Z* = 21.60, N_fs0.05_ = 161.47, N_fs0.01_ = 73.94) indicated that the pooled ORs were stable in our meta-analysis.

## Discussion

Previous studies have demonstrated that epigenetic alteration is an important event in the carcinogenic progression. Particularly, increased methylation in the promoter region of tumor suppressor gene can account for a progressive reduction of its expression, silencing and selective proliferative advantage in certain cells, which plays a vital role in the development of human cancer^39^. The aberrant methylation has been observed in the promoter region of *RASSF1A* in various cancers, including HNSCC^6^.

Our meta-analysis included 550 HNSCC tissues and 404 controls from 12 published studies. Overall, the pooled OR of *RASSF1A* methylation in cancer tissues and controls under the random-effects model was 2.93 (95% CI: 1.58–5.46), which suggested a significant association of the methylation of *RASSF1A* promoter with HNSCC. The overall heterogeneity between included studies was interpreted by the *χ*^2^-based Cochran *Q* statistic test and *I*^*2*^ statistics and meta-regression was used to explore the sources of the heterogeneity. When *I*^2^ > 50% and *P* < 0.1 for the Q statistic, the between-study heterogeneity was considered significant and the pooled ORs was calculated using a random-effects model (the DerSimonian-Laird estimate)^40^. Otherwise, a fixed-effects model (the Mantel-Haenszel test) was applied^40^. In the subgroups of races, the pooled ORs were 3.45 (95% CI: 1.85–6.44) in Caucasians subgroup under the fixed-effects model and 2.75 (95% CI: 0.96–7.89) in Asians subgroup under the random-effects model, respectively. This indicated that hypermethylation of *RASSF1A* had a stronger association with increased risk of HNSCC in Caucasians. Similarly, the methylation rates of the MGMT gene and GSTP1 gene in non-small cell lung cancer were also significantly higher in Caucasians than in Asians^41^, and this divergence might be due in large part to a combination of differences in allele frequencies and complex epistasis or interactions between the gene and environment^42^. The summary OR was 3.19 (95% CI: 1.30–7.83) in the autologous control subgroup under the random-effects model, and was 2.52 (95% CI: 1.35–4.73) in the heterogeneous control subgroup under the fixed-effects model. Interestingly, this was consistent with a previous study of non-small cell lung cancer (NSCLC)^43^, which indicated an increased likelihood of *RASSF1A* methylation in heterogeneous controls compared to autologous controls. The reason for this might be because the benign lesions had a higher probability of *RASSF1A* methylation as an early stage of carcinoma. In the method subgroup, the ORs were 10.76 (95% CI: 0.56–211.78) in the BSP group, 1.06 (95% CI: 0.38–2.94) in the Q-MSP group, and 3.30 (95%CI: 2.17–5.01) in the MSP group under the fixed-effects model. The differences of ORs in these subgroups were potentially caused by the different sensitivities and specificities of the method used to the detection of gene methylation. Q-MSP is a sensitive quantitative assay with normalization of the amplifiable DNA content of samples. The cut-off point of Q-MSP was derived from the best distinguish point. However, the cut-off point of MSP is defined by visual detection of the presence or absence of PCR product compared to the intensity of controls^44^. Therefore, MSP (a nonquantitative and nonfluorometric method) would be hard to detect low levels of promoter methylation, while Q-MSP can detect up to 1/1000 methylated alleles^45^ and this would have an impact on the results. BSP, a method of genomic sequencing, can provide a more direct and quantitative analysis for most CPG sites within a defined region than MSP and Q-MSP^46^. The 95% confidence intervals of ORs of BSP method subgroup and Q-MSP method subgroup included 1. This was potentially attributed to the relatively small sample size (less than 60) in these subgroups. The pooled OR also differed according to different sample size. In the <40 cases subgroup, the OR was 2.30 (95% CI: 1.25–4.21) under the fixed-effects model. In the ≥40 cases subgroup, the OR 3.87 (95% CI: 1.46–10.25) under the random-effects model. However, there was no significant difference between different sample size subgroups.

Some potential limitations of the study should be taken into consideration when interpreting the results of meta-analysis. Firstly, due to the 12 included studies were retrospective, there might be a potential unidentified confounding bias, information bias and selection bias. Secondly, although we explored and evaluated the source of heterogeneity in four variables, we could not explore heterogeneity from other aspects because of the insufficient demographic and clinical data. Thirdly, previous studies demonstrated that time of sampling^47^ and fixation techniques^48^ potentially influenced methylation status in paraffin-embedded tumors. The 12 included studies varied in time of sampling and fixation techniques and these could result in heterogeneity. Additionally, since the number of included studies and samples were relatively small, further investigations with a large number of samples were required.

## Conclusions

Our meta-analysis identified an association between aberrant rmethylation of *RASSF1A* promoter with HNSCC, which indicated that hypermethylation of *RASSF1A* promoter might be a potential biomarker in the process of HNSCC. Prospective studies with larger sample size are needed to confirm these results in the future.

## Methods

The meta-analysis was performed according to the latest meta-analysis guidelines (PRISMA).

### Studies identification

Studies were identified via an electronic search of a range of computerized databases, including PubMed, Embase, Web of Science and CNKI using the following key words: ‘squamous cell carcinoma or cancer’, ‘oropharyngeal or oropharynx or head and neck or tonsil’, ‘RAS association domain family protein 1A’, ‘*RASSF1A*’, ‘methylation’ and ‘hypermethylation’. Articles were searched in the databases form Jan 1, 2000 to May 8, 2015 without language limitation. Two independent reviewers screened the titles and abstracts identified by the electronic search to identify relevant studies. The inclusion criteria of the meta-analysis were as follows: (1) case-control study design; (2) presentation of data necessary for calculating odds ratios (ORs); (3) studies primarily evaluating the incidence of *RASSF1A* methylation in HNSCCs and corresponding autologous/heterogeneous control, including non-tumor tissue, plasma and sputum of HNSCC patients. The excluded studies were as follows: duplication, review, animal study, experimental study and adequate specific data.

### Data extraction

Data retrieved from the eligible studies including first author’s name, year of publication, published journal and country, patient ethnicity, population size, methods used to determine methylation status, histology, control type, and methylation status of *RASSF1A* promoter in extracted cancer tissues and controls. Data extraction was conducted by two reviewers independently using a standard data extraction form. If there were disagreement between them, a third reviewer was used to reach a consensus.

### Statistical analysis

All statistical analyses were conducted by using the Meta package (version 2.2-1) in R (version 3.0.2; http://www.r-project.org/). The pooled odds ratios (ORs) of different studies and corresponding 95% confidence intervals (CIs) were calculated to evaluate the strength of the association between *RASSF1A* methylation and HNSCC risk. In order to assess the percentage of variability across studies attributable to heterogeneity beyond by sampling error, the χ^2^-based Cochran Q statistic test and *I*^2^ statistics were employed. When *I*^2^ > 50% and *P* < 0.1 for the Q statistic, the between-study heterogeneity was considered significant and the pooled ORs was calculated using a random-effects model (the DerSimonian-Laird estimate)^40^. Otherwise, a fixed-effects model (the Mantel-Haenszel test) was applied^40^. If the heterogeneity was significant, to explore and assess the source of heterogeneity, a meta-regression (restricted maximum-likelihood estimator method, REML^35^) was initially performed and a subgroup analysis was followed according to the results of meta-regression. Sensitivity analysis was employed to assess the effects of single study on pooled ORs after omitting one study. Publication bias was assessed by a funnel plot for Egger’s test. When the individual studies had cells with zero counts, the default was to add 0.5 to all zero counts in the Meta package. Statistical significance was defined as a two-tailed *P* value of 0.05 in our study.

## Additional Information

**How to cite this article**: Meng, R.-W. *et al.* Aberrant Methylation of *RASSF1A* Closely Associated with HNSCC, a Meta-Analysis. *Sci. Rep.* **6**, 20756; doi: 10.1038/srep20756 (2016).

## Figures and Tables

**Table 1:** General Characteristics of the Included Studies.

First author	Year	Location	Race	Mean/median age (range) (y)	Gender (M/F)	Case (n)		Control (n)		Method	Control style	Control source
						M+	U	M+	U			
Hogg^34^	2002	UK	Caucasians	NA	NA	4	24	0	24	BSP	A	NMT
Dong^26^	2003	USA	Caucasians	NA	NA	7	46	0	46	MSP	A	ANT
Maruya^22^	2004	USA	Caucasians	58 (31–81)	26/6	0	32	1	32	MSP	A	NMT
Xu^23^	2006	China	Asians	60 (41–76)	35/13	34	48	11	48	MSP	A	ANT
Righini^27^	2007	French	Caucasians	57 (33–74)	NA	14	90	0	30	MSP	A	NMT
Wan^31^	2007	China	Asians	NA	17/15	13	32	4	28	MSP	H	OCT
								0	10	MSP	H	OCT
Ghosh^33^	2008	India	Asians	NA	NA	23	111	9	52	MSRA	H	DLT
Steinmann^28^	2009	Germany	Caucasians	57 (41–77)	NA	10	54	0	23	MSP	A	CMT
Su^24^	2010	Taiwan	Asians	55 (37–82)	47/5	9	31	12	31	Q-MSP	A	ANT
								0	12	Q-MSP	H	NMT
Laytragoon-Lewin^29^	2010	Sweden	Caucasians	62 (42–101)	30/11	8	18	4	18	MSP	A	NMT
Paluszczak^30^	2011	Poland	Caucasians	58 (41–75)	35/6	13	41	9	41	MSP	A	NMT
Koutsimpelas^31^	2012	Germany	Caucasians	62 (45–83)	19/4	3	23	0	3	MSP	H	GT

Abbreviation: NA, not available; M, male; F, female; M+, methylated; U, unmethylated; A, Autologous (the control from the HNSCC patients themselves); H, Heterogeneous (the control from other individuals); NMT, normal mucosa tissue; ANT, adjacent non-tumor tissue; OCT, oral cavity tissue; DLT, dysplastic lesions tissue; CMT, cheek mucosa tissue; GT, gingiva tissue.

**Table 2:** Meta-regression analysis.

Heterogeneity sources	Coefficient	95%CI		*P*
		Lower	Upper	
Races	−1.22	−2.58	0.14	0.08
Control types	−0.19	−1.46	1.07	0.76
Methods				
MSP	−1.78	−5.29	1.73	0.32
Q-MSP	−3.42	−7.19	0.35	0.08
Case sample size	0.38	−1.02	1.78	0.59

Races: Asians and Caucasians; Control types: autologous control and heterogeneous control; Methods: MSP (methylation-specific polymerase chain reaction), Q-MSP (quantitative methylation-specific polymerase chain reaction) and BSP (bisulfite sequencing polymerase chain reaction); Case sample size: <40 and ≥40.

**Table 3:** Subgroup analysis of the association between *RASSF1A* and HNSCC.

Group	Case		Control		M-H pooled OR^*^	D+L pooled OR^†^	Heterogeneity		
	M+	U	M+	U	OR (95% CI)	OR (95% CI)	*I*^*2*^(%)	*P*	τ^2^
Total	138	550	50	404	2.93 (2.01–4.26)	**2.93 (1.58**–**5.46)**	46.7	0.0372	0.4669
Races									
Asians	79	222	36	181	2.63 (1.64–4.22)	**2.75 (0.96**–**7.89)**	77.6	0.0039	0.8887
Caucasians	59	328	14	223	**3.45 (1.85**–**6.44)**	2.83 (1.35–5.93)	6.9	0.0869	0.3772
Control types									
Autologous	99	384	37	293	3.13 (2.00–4.90)	**3.19 (1.30**–**7.83)**	59.6	0.0112	0.9318
Heterogeneous	95	309	13	111	**2.52 (1.35**–**4.73)**	2.69 (1.06–6.87)	29.7	0.2236	0.3298
Methods									
BSP	4	24	0	24	**10.76 (0.55**–**211.78)**	10.76 (0.55–211.78)	—	—	—
Q—MSP	9	31	12	43	**1.06 (0.38**–**2.94)**	1.06 (0.38–2.94))	—	—	—
MSP	125	495	28	337	**3.30 (2.17**–**5.01)**	3.27 (1.67–6.40)	44.7	0.0615	0.442
Case sample size									
<40	37	160	21	164	**2.30 (1.25**–**4.21)**	2.28 (1.00–5.20)	26.1	0.2389	0.2678
≥40	101	390	29	240	3.36 (2.07–5.43)	**3.87 (1.46**–**10.25)**	62.7	0.0199	0.7714

Abbreviation: *RASSF1A*, RAS association domain family protein 1a; HNSCC, head and neck squamous cell carcinoma; BSP, bisulfite sequencing polymerase chain reaction; Q-MSP, quantitative methylation-specific polymerase chain reaction; MSP, methylation-specific polymerase chain reaction; M+, methylated; U, unmethylated.

^*^The fixed-effects model.

^†^The random-effects model.

The numbers with bold font were the results under the model applied to calculate the pooled ORs. When *I*^2^ > 50% and *P* < 0.1 for the Q statistic, the pooled ORs was calculated using a random-effects model. Otherwise, a fixed-effects model was applied.
